# Advances in HBV infection and replication systems *in vitro*

**DOI:** 10.1186/s12985-021-01580-6

**Published:** 2021-05-29

**Authors:** Ruirui Xu, Pingping Hu, Yuwen Li, Anran Tian, Jun Li, Chuanlong Zhu

**Affiliations:** 1 Department of Infectious Disease, The First Affiliated Hospital of Nanjing Medical University, Nanjing, 210029 Jiangsu China; 2 Department of Pediatrics, The First Affiliated Hospital of Nanjing Medical University, Nanjing, 210029 Jiangsu China; 3 Department of Tropical Diseases, The Second Affiliated Hospital of Hainan Medical University, Haikou, 570311 Hainan China

**Keywords:** Cell culture, HBV, NTCP, Virus-host interactions, 3D cell culture system

## Abstract

**Background:**

Hepatitis B virus (HBV) is a DNA virus belonging to the Hepadnaviridae family that has limited tissue and species specificity. Due to the persistence of HBV covalently closed circular DNA (cccDNA) in host cells after HBV infection, current antiviral drugs cannot eradicate HBV. Therefore, the development of an active cell culture system supporting HBV infection has become the key to studying HBV and developing effective therapeutic drugs.

**Main body:**

This review summarizes the significant research achievements in HBV cell culture systems *in vitro*, including embryonic hepatocytes and primary hepatocytes, which support the virus infection process most similar to that in the body and various liver tumor cells. The discovery of the bile-acid pump sodium-taurocholate co-transporting polypeptide (NTCP) as the receptor of HBV has advanced our understanding of HBV biology. Subsequently, various liver cancer cells overexpressing NTCP that support HBV infection have been established, opening a new door for studying HBV infection. The fact that induced pluripotent stem cells that differentiate into hepatocyte-like cells support HBV infection provides a novel idea for the establishment of an HBV cell culture system.

**Conclusion:**

Because of the host and tissue specificity of HBV, a suitable *in vitro* HBV infection system is critical for the study of HBV pathogenesis. Nevertheless, recent advances regarding HBV infection *in vitro* offer hope for better studying the biological characteristics of HBV, the pathogenesis of hepatitis B, the screening of anti-HBV drugs and the mechanism of carcinogenesis.

## Introduction

Hepatitis B virus (HBV) is a partially circular double-stranded DNA virus with a limited host range and high species specificity. Generally, only the liver tissue of humans, chimpanzees, and tree shrews are susceptible to HBV [1]. HBV infection can cause hepatitis and liver dysfunction, and the disease can progress to chronic infection, with some further progressing to cirrhosis or even liver cancer. It is also a severe global health problem worldwide, especially in Asia and Africa. According to the latest global epidemiological statistics regarding HBV, an estimated 257 million people are living with HBV infection (defined as hepatitis B surface antigen-positive). Approximately 1 million people die each year from liver failure, cirrhosis or primary hepatocellular carcinoma caused by HBV infection, and approximately 300,000 of these individuals reside in China. HBV is the leading cause of death among all infectious diseases. Due to the persistence of HBV covalently closed circular DNA (cccDNA) in host cells after HBV infection, antiviral drugs cannot eradicate HBV [2–4], and a safe and curative treatment for patients with chronic HBV infection is therefore lacking. Currently, establishing an effective HBV infection system is the key to studying viral infection mechanisms and drug screening to solve this problem. However, because of the loss of HBV receptors on the surface of hepatocytes and changes in the cell structure and growth environment under *in vitro* conditions [5], establishing a stable hepatic-derived HBV infection system *in vitro* is difficult. Currently, many HBV infection systems *in vitro* have been established in the field of hepatitis B research. Although these systems have shortcomings, they are useful in the study of HBV to some extent and play an important role in the development and evaluation of anti-HBV drugs.

This review summarizes representative HBV *in vitro* infection systems, including recombinant cell lines obtained by integrating the HBV genome into the liver cancer cell genome by genetic engineering techniques and sodium-taurocholate co-transporting polypeptide (NTCP) overexpressing hepatoma cell lines permissive for HBV infection established based on the discovery of the HBV-specific receptor bile-acid pump NTCP. Besides, the differentiation of induced pluripotent stem cells into hepatocyte-like cells (HLCs) provides more possibilities for studying HBV. The establishment of the HBV/hepatitis C virus (HCV) coinfection system provides a reliable platform for studying the interaction between HBV and HCV and the host.

## HBV replication cell lines

### HepG2.2.15 cells

Sells et al. introduced the recombinant vector pDoLT-HBV-1 (a vector that contains two head-to-tail dimers of HBV in a tail-to-tail orientation) and a plasmid containing the neomycin resistance gene into the human hepatoma cell line HepG2 by co-transfection and established the HepG2.2.15 cell line by G418 screening [6]. The cell line carries HBV DNA that includes gene sequences integrated into the host chromosomal, extrachromosomal relaxed circular DNA, cccDNA and an incomplete copy of the HBV genome. Besides, the cell line can produce a variety of HBV-specific mRNAs (3.5 kb, 2.5 kb, 2.1 kb) [7] and express all viral markers, stably secreting HBsAg, HBeAg and Dane particles for a long period. The concentration of HBsAg detected in the culture supernatant of HepG2.2.15 cells reached 4.2~94.3 µg/L, and 22 nm spherical and rod-shaped particles as well as 42 nm particles could be detected by immunoelectron microscopy, which confirmed that HepG2.2.15 cells could support not only the replication of HBV DNA but also the packaging and secretion of Dane particles. Since this cell line was derived from hepatoma cells, it can be subcultured for a long time. Besides, it supports continuous virus replication and produces infectious virions, so it is a widely used cell culture system for studying HBV. Although the establishment of the HepG2.2.15 cell line provides an effective model for studying the structure, function, gene expression and regulation of HBV DNA and the initial screening of anti-HBV drugs *in vitro*, this cell line also has certain limitations, including the following. (i) This cell line does not recapitulate natural infection: the HBV DNA is integrated into the chromosome of the host cell, so it can simulate the process of virus replication but not the process of virus invasion into cells.(ii)This cell line is insensitive to direct infection with serum containing HBV, which due to the lack of NTCP, a specific receptor for HBV infection.(iii) Although the replication and expression of HBV in hepatocytes are reproduced, this model is divorced from the environment in which the body's immune system affects HBV (iv) HepG2.2.15 cells cannot be used for the study of HBV adsorption, cellular entry or virus uncoating. (v) Since HepG2.2.15 cells were derived from HepG2 cells, they cannot be used for the study of HBV carcinogenicity. This cell line has been used in studies on the later steps of the HBV life cycle, the interaction of immune cells with cells containing HBV, and the evaluation of antiviral drugs.

### HepAD38 (EF9, EFS19) cells

Ladner et al. transfected HepG2 cells with the plasmid pTet-HBV which was constructed by removing the cytomegalovirus immediate-early (CMV-IE) promoter from plasmid pCMV-HBV fused with the ayw subtype of the HBV genome and replacing it with the tetracycline-responsive CMV-IE promoter to obtain the HepAD38 cell line [8]. The HepAD38 cell genome contains 1.1 copies of the HBV genome, whose expression is regulated by the inducible CMV-IE promoter. Due to the disruption of the precore gene, the HepAD38 cell line produces approximately 11 times more HBV DNA than HepG2.2.15 cells. In the HepAD38 cell line, tetracycline can be used to regulate HBV replication. When tetracycline is contained in the medium, HBV cannot be synthesized because of the inhibition of pgRNA synthesis. After removing tetracycline, the cells immediately express pgRNAs, cccDNA and HBV. Owing to the low sensitivity of direct cccDNA detection and the fact that the detection results are susceptible to interference by rcDNA signals, the HBeAg secreted by HepAD38 cells can be used as the main surrogate marker of cccDNA; therefore, the replication level of cccDNA can be estimated by detecting HBeAg directly. Compared to HepG2.2.15 cells, HepAD38 cells produce higher levels of HBV and can accurately regulate the commencement of viral replication. Similar to that of HepG2.2.15 cells, the limitation of the HepAD38 cell line is that it is not suitable for studying the interaction between virus and host cells in the early stage of HBV infection. This HBV cell culture system is suitable for studying HBV replication processes and anti-HBV drug screening. Guo et al. and Cai et al. optimized HepAD38 cells to generate HepDE19 and HepDES19 cells. HepDE19 cells perform all the functions of HepAD38 cells, but the dependency relationship between secreted HBeAg and cccDNA is closer than that in the HepAD38 cell line; thus, HBeAg is the only surrogate marker of cccDNA. While HepDES19 cells produce more cccDNA than HepDE19 cells, HepDES19 cells are more suitable for screening anti-HBV drugs and for observing the effects of drugs on cccDNA [9, 10]. In addition, Guo H and his colleagues established HepBHAe82 cells, which improved the detection of cccDNA marker [11]. Another derivative, Hep38.7-Tet cells, which have higher HBV replication and cccDNA levels than the abovementioned cell lines, has also been used [12].

### Ad-HBV1.3 system

He et al. used adenovirus as a vector to introduce a 1.3-fold overlength HBV genome into the 293packaging cell line and then infected HepG2 cells with packaged recombinant virus (Ad-HBV1.3) to construct the Ad-HBV1.3-HepG2 system. This system can effectively initiate the replication of hepatitis DNA virus and express a high level of HBV. HBV protein, RNA, DNA and all replicative intermediates can be detected among the products. The presence of cccDNA indicates that intracellular hepadnavirus replication takes place in the native transcriptional template outside the chromosome; therefore, the replication cycle is independent of linear viral genomes, same as in natural infections [13]. The adenoviral vector can be used to adjust the level of HBV replication by altering the amount of recombinant viral DNA. Adenovirus-mediated HBV genomic transfer can help to study the ability of cells from different species to support HBV replication and the role of viral proteins in regulating the viral life cycle. Because of the integration of the green fluorescent protein (GFP) gene into the adenovirus cytoskeleton plasmid, the infection efficiency of the virus can be directly observed. After adenovirus genome transfer, the establishment of hepadnavirus cccDNA in heterologous cells will be helpful to investigate which step of the hepadnavirus replication cycle is supported by each cell and to analyze the cellular determinants [13]. Unlike the baculovirus system, in which gene transfer is limited to certain species [14], this system has no species barrier. Compared with the HepG2.2.15 cell line, this system has the advantages of highly expressing HBV and artificially controlling the mutation and expression of the HBV genome and can be used for *in vivo* experiments. In particular, of all known gene delivery vectors, adenovirus vectors are the most effective for transferring exogenous DNA to the livers of various experimental animals [15, 16]. The Ad HBV system has no species barrier, so it can achieve HBV replication in hepatocytes of its non-specific host. However, this system has significant cytotoxicity, which might restrict its application on certain questions, such as due to the failure of an increased vector dosage to increase antigen production, it is not suitable for assessing the antiviral effects of drugs.

### HBV baculovirus system

Delaney et al. used baculovirus to introduce a replication competent HBV genome into HepG2 cells to establish the HBV recombinant baculovirus/HepG2 system [17]. The recombinant system can express various HBV antigens. High levels of HBV antigen, replicative intermediate, extracellular DNA, and cccDNA can be detected in this system [18]. In addition to secreted antigens, viral products such as HBV transcripts, replicative intermediates, and cccDNA were also present at levels proportional to the multiplicity of infection (MOI). HBV replication in the HBV recombinant baculovirus/HepG2 system can be maintained at high levels for at least 35 days with a dose-dependent expression level and virus infection. Compared with HepG2.2.15 cells, this cell line has an approximately 100 times higher HBV replication level. A unique aspect of the HBV recombinant baculovirus/HepG2 system is its ability to easily detect rcDNA and cccDNA; therefore, the system can be used to quantify the effects of antiviral agents on nuclear HBV DNA [19]. It can also be used to study the resistance of HBV to nucleoside analogs [17, 18]. However, the HBV recombinant baculovirus/HepG2 system also has flaws: (i) Baculovirus enters mammalian cells through nonspecific endosomal uptake rather than receptor-mediated mechanism [20]; (ii) baculovirus-mediated gene transfer is restricted to certain species; and most importantly, (iii) traditional baculovirus vectors are not suitable for use in animal experiments because they are rapidly inactivated by the complement system [14, 21].

## Cell lines that can be infected with HBV

### Human fetal hepatocytes

As the natural host of HBV, human embryonic hepatocytes have characteristics similar to those of adult hepatocytes; therefore, many researchers initially tried to establish and apply the human embryonic hepatocyte system. Ochiya et al. obtained large numbers of mononuclear polyhedral hepatocytes arranged in trabeculae from fetal liver tissue at 20-24 weeks of gestation. After cultured for approximately one week, glycogen, glucose-6-phosphatase and transpeptidase could be detected in the medium; on the second day after the cells were plated, albumin could be detected and continued to be secreted until the 16th day, retaining the regular morphologies and biochemical characteristics for at least 2 weeks of subsequent culture [22]. Viral replication indexes were detected both in the culture medium and intracellularly after using primary human fetal hepatocytes with HBV infection. It is clear that fetal human hepatocyte culture *in vitro* can simulate the biological function of hepatocytes in the human body, and the cells have enhanced survivability, proliferation and differentiation compared with human primary adult hepatocytes [22]. Its advantages as a replication system of HBV infection include the following: (i) the cells can be infected by serum containing HBV particles; (ii) all known viral proteins, RNAs and DNAs observed in HBV infected livers *in vivo* can also be produced in infected fetal human hepatocytes *in vitro*; (iii) the cells release infectious viral particles; (iv) the cells can produce cccDNA. However, this system also has limitations; its infection efficiency to HBV is only approximately 12%; moreover, after being attacked by the virus, the cells are no longer sensitive to HBV, and thus the spreading of virus to adjacent cells does not occur [22]. Productive infection of these cells can be maintained for only a limited period of time (up to 16-18 days), while maintaining a normal hepatocyte phenotype [23]. Lázaro et al. established the serum-free primary cultures of human fetal hepatocytes that can retain hepatocytic traits for 2-4 months [24]. Zhou et al. cocultured primary embryonic hepatocytes with nonparenchymal hepatocytes to induce hepatocyte islands, enhancing the differentiation ability of fetal hepatocytes. This resulted in the maintenance of liver function for up to 3 months and maintenance of susceptibility to HBV infection for 10 weeks under *in vitro* culture conditions [25]. However, the long-term culture of isolated human embryonic hepatocytes *in vitro* and the preservation of stable biological characteristics remain a problem. The ability of cells to differentiate is limited, and some hepatocyte functions are quickly lost. Importantly, the optimized model could still not overcome the rapid decrease in susceptibility to HBV under *in vitro* culture conditions. In addition, the limited availability of fetal hepatocytes and donor-dependent variations are major limitations of this system. This model is suitable for studying the early stage of HBV infection [22].

### Adult human hepatocytes

The study of HBV-host cell interactions requires an appropriate and reproducible tissue culture system to reliably mimic the viral life cycle, and this need has prompted many researchers to focus on the establishment of *in vitro* systems by a variety of approaches. However, no effective cell culture system has thus far been developed to support this research. Cultures of primary human adult hepatocytes have the most similar physiological characteristics to hepatocytes *in vivo*, and thus it is an ideal model for studying HBV. High-yield and high-activity primary human hepatocytes were obtained by two-step perfusion using liver tissue surgically removed from a patient’s liver lobe. *In vitro* infection experiments confirmed that the cells could be naturally infected by HBV, thereby providing a better method for selecting the tissue source for primary human hepatocyte culture and establishing an HBV infection system [26]. Rijntjes et al. demonstrated that normal primary human hepatocytes can be cryopreserved for a long time. These cells can survive and maintain their typical cell phenotype for 3–4 weeks when inoculated onto an artificially prepared extracellular biological substrate after thawing [27]. Gripon et al. inoculated primary human adult hepatocytes with human serum containing HBV-infected particles, and the detection of HBV antigen and HBV DNA in the culture supernatant indicated that HBV could infect the primary adult hepatocytes [5]. Galle et al. reported that adult hepatocytes seeded on collagen gels after isolation could maintain cell viability for 4 to 6 weeks. Freshly isolated and plated adult hepatocytes were inoculated with human serum containing 10^12^ HBV-infected particles per liter (1:20 or 1:200 dilution). The results showed that high levels of HBsAg and low levels of HBeAg were secreted in the culture supernatant on the 6th day after infection, reaching maximum values on the 12th day and thereafter declining after 14 days, which indicated HBV replication [28]. Subsequently, Schulze-Berga et al. improved the culture method to prolong the growth time of primary adult hepatocytes *in vitro*, while maintaining their proliferation capacity and liver-specific functions [26].

Katsura et al. used keratinocyte growth factor (KGF) medium, adding 10% human serum, 10 mol/L nicotinamide (VPP), 10 μg/L endothelial cell growth factor (ECGF), 0.5 mg/L insulin, and 10^-7^ M dexamethasone as the basic culture medium for human primary hepatocytes, which prolonged their survival time to 56 days and simultaneously maintained their differentiation and function [29]. Gripon et al. reported that HBV infection was greatly enhanced when adult primary hepatocytes were coincubated with HBV in the presence of polyethylene glycol (PEG) [30]. Notably, Ishida et al. established a novel HBV infection system *in vitro* using fresh human hepatocytes isolated from the chimeric mice with humanized liver, which demonstrated susceptibility to HBV, and the maximum infection rate was approximately 80% in the presence of PEG. Besides, this system can support the complete HBV life cycle [31]. Ulvestad et al. simulated the microenvironment of the liver by allowing human hepatocyte cultures to be maintained for a long period and to retain numerous liver-specific functions by culturing human primary hepatocytes in a 3D bioreactor system [32]. These findings have laid the foundation for studying the pathogenesis of HBV and screening antiviral drugs using the primary hepatocyte model. However, although HBV infected adult primary hepatocytes similar to HBV natural infection, the cells could not be subcultured and the growth time was limited. Additionally, after plating, the function of mature hepatocytes declines rapidly and the cells lose their typical polygonal morphology, causing the gradual loss of sensitivity to viruses, which is the main obstacle to their application. This may be because hepatocytes cultured *in vitro* lose cell-cell communication between parenchymal and nonparenchymal cells in an *in vivo* environment, and their interaction is essential for regulating cell growth and differentiation and for coordinating the multiple functions of the liver [33–35]. Primary human hepatocytes have advantages that no tumor cell line can match, which include direct infection by HBV, close resembling the physiological and biological indicators of natural infection, making primary human hepatocytes the most reliable *in vitro* infection system.

However, primary hepatocytes are terminally differentiated cells that cannot be subcultured and have a limited life cycle. The rapid loss of the unique function and morphology of mature liver cells leads to the gradual loss of susceptibility to HBV. Although the problem of primary human hepatocyte source scarcity and the inability to scale up in previous years has limited the application of primary human hepatocytes in related fields, some laboratories have recently reported methods of primary human hepatocyte amplification *in vitro* to solve this problem. In the Yan He-Xin laboratory, the 2D culture method was used to induce human hepatocytes to dedifferentiate into liver stem cells that could be expanded *in vitro*, thereby reversing and expanding primary hepatocytes and leading to the development of a new cell source for HBV-host cell interaction studies [36]. Using the 2D culture method, Zhang et al. added Wnt3a and other factors to the culture medium to establish a new *in vitro* culture system for human hepatocytes, which increased the amplification of human primary hepatocytes *in vitro *by up to 10,000-fold [37]. Recently, the Roel Nusse laboratory at Stanford University and the Hans Clevers laboratory in the Netherlands successfully expanded human primary hepatocytes *in vitro* by inducing hepatocytes to form organoids *in vitro* [38, 39]. Although the various methods for culturing human primary hepatocytes have their own advantages and disadvantages, the establishment of these methods for the *in vitro* expansion of hepatocytes will certainly substantially promote the development of liver research, enabling many experiments that were previously impossible. Primary human hepatocytes are commercially available.

Compared with the traditional 2D monolayer cell culture, 3D cell culture has significant advantages. 3D cell-culture models exceed 2D culture systems by promoting higher levels of cell differentiation and tissue organization. 3D culture technology creates a three-dimensional micro-environment for liver cells, which can accurately reproduce the complex environment of liver cells in natural tissues *in vitro*, and achieve a high degree of simulation of the real ECM (extracellular matrix) of biological tissues *in vitro*. In recent years, various liver 3D models have been proposed, including 3D liver ball models, liver slice systems based on microfluidic technology, etc., and their culture methods and materials used are different.

PHH can be cultured as 3D spheroids, with diameters between 200 and 300 µm. Multiple methods for the generation of spheroids have been presented, including stirring bioreactors [40], aggregation in hanging drops, or culture on ultralow attachment (ULA) surfaces. 3D spherical cultured hepatocytes retained their RNA expression levels of various phase I (CYP1A2, CYP2C9, and CYP3A4) and phase II enzymes (GSTA1 and UGT2B7) [41]. Immunofluorescence microscopy of human hepatocyte spheroids confirmed the presence of the liver-specific markers, hepatocyte nuclear factor 4α, albumin, cytokeratin 18, and cytochrome P450 3A. In addition, hepatocyte spheroids can spontaneously assemble a functional bile canaliculi network after 3-4 weeks of culture [40]. PHH spheres retain the drug metabolism and metabolic characteristics of freshly isolated hepatocytes, which can be used for long‐term analyses of drug metabolism and liver function and moreover is suitable for investigating *in vitro* metabolism of very low clearance drugs as well as for studying time‐dependent inhibition of drug metabolism for relevant periods [42]. In addition to determining drug clearance, they are used for metabolite identification, enzyme inhibition, and enzyme induction studies [43–45].

As these models are static and closed, the concentration of metabolites will rise to non-physiological levels, and the compact structure of 3D cultured cells will cause hypoxia and nutrient transport problems.

The liver chip based on microfluidic technology can effectively solve these problems. Microfluidics is a technology that precisely controls and manipulates micro-scale fluids, especially sub-micron structures. It is also called Lab-on-a-Chip or microfluidic chip technology. The application of microfluidics in organs-on-chips enables the efficient transport and distribution of nutrients and other soluble cues throughout the viable 3D tissue constructs. The liver-chip based on microfluidic technology provides physiologically relevant conditions that can retain the *in vivo*-like phenotype and bioactivity of hepatocytes [46]. The Advantages of microfluidic cell culture systems include the presence of dynamic flow conditions and mechanical stimulations within their microchannels, mimicking what is observed *in vivo* [47]. Furthermore, microfluidic devices can provide chemical concentration gradients with high sensitivity and precision. These gradients are indispensable for regulating essential biological processes such as chemotaxis, cell migration and differentiation, immune responses, and wound healing.

### Co-culture system

Although primary hepatocyte culture is the best model for studying HBV infection *in vitro*, in most studies, within a few days of being isolated, primary human hepatocytes undergo a rapid dedifferentiation process and viral infections are abortive due to the rapid loss of hepatic functions [28].

Zhou et al. established a feasible method to prevent this dedifferentiation by co-culturing human fetal hepatocytes with hepatic non-parenchymal cells to maintain the differentiation features of human fetal hepatocytes [25]. In this co-culture system, the bile canalicular structures could be observed and hepatocytic features could be further maintained for up to an additional 3 months. Morphological examination showed that the piled-up hepatocytes formed island-like aggregates, and the piled-up hepatocytes in the 'hepatic islands' were surrounded and invaded by non-parenchymal cells. There are a number of multiple cellular cavities formed by an orderly arrangement of albumin-positive hepatocytes in the culture, which were probably liver organoids. Both albumin and CK18, which are the markers of mature hepatocytes, were stably expressed in this co-culture system. CYP 3A4, a member of the cytochrome P450 mixed-function oxidase system, was also expressed at high levels throughout the culture period, indicating good maintenance of the drug-metabolizing ability in the cocultured hepatocytes. Infecting these cultures with HBV, the infected hepatocytes survived, and continued to secrete HBsAg and HBeAg up to 114 days post-seeding, and cccDNA was also observed in the cells infected with HBV. Most importantly, these human fetal hepatocytes still exhibited susceptibility to HBV infection after long-term maintenance, for as long as 10 weeks.

Winer et al. established SACC by plating PHHs with non-parenchymal stromal cells in collagen-coated tissue culture plates, utilizing reported protocols to promote advanced liver morphology, to enhance many liver specific functions in order to extend the culture periods [48, 49]. HBV infection in SACC PHH was highly reproducible and did not depend on particular lots of pooled hepatocyte donors or batches of cell culture-derived HBV inocula. HBsAg, HBeAg, cccDNA and pgRNA were detected in SACC-PHHs infected with HBV. Immunofluorescent visualization of HBcAg demonstrated that most of the hepatocytes in the culture were infected. The secretion of HBsAg sustained for more than 30 days postinfection without suppression of cell-intrinsic antiviral defenses. When HBV was used to infect SACC PHH prepared from hepatocytes of different donors, only minor differences in the quantity of cccDNA and pgRNA were observed, indicating that SACC-PHHs were robustly infected. Therefore, the platform could be scaled to a format amenable to high throughput screening (HTS)applications. Moreover, the SACC-PHH platform can be used to test the utility of various direct-acting antivirals (DAAs) and putative host-targeting antivirals (HTAs). The SACC-PHHs platform may have utility for assessing preclinically the efficacy of other entry inhibitors and possibly (vaccine-induced) neutralizing antibodies [50].

### Primary Tupaia hepatocytes

Tree shrews are small nonchewing toothed animals similar to primates in terms of phylogeny. They are the only animals known to be infected with HBV other than chimpanzees. HBV can infect primary tree shrew hepatocytes. cccDNA and four kinds of mRNA can be detected in cultured hepatocytes, and secretion of HBsAg and HBeAg can be detected in the cell culture supernatant [51]. The early phase of HBV infection of tree shrew hepatocytes is very similar to that of human hepatocytes, in which the pre-S1 and S antigens are essential [52]. However, the infection efficiency of tree shrew liver cells by HBV is low. Studies have shown that human serum components can block HBV infection of tree shrew liver cells, while purified virus particles can significantly enhance the ability of the virus to bind and infect tree shrew hepatocytes. To eliminate the effect of human serum components on viral invasion, Yan et al. infected tree shrew hepatocytes with recombinant adenovirus vector containing the whole HBV genome, and the cultured primary tree shrew hepatocytes could support all processes of HBV replication. In addition to forming cccDNA and secreting HBsAg and HBeAg, the cells could also support the generation of complete virus particles. This system has some advantages over other cell culture systems:(i) primary Tupaia hepatocytes are more readily available and exhibit a more constant susceptibility to HBV than primary human hepatocytes; and (ii) the results of infecting primary Tupaia hepatocytes with HBV *in vitro* can be verified *in vivo* by infection of Tupaia with HBV. Tree shrew primary hepatocytes have been widely used to study HBV infection. In a study by Yan H et al., the original Tupaia hepatocytes were used as target cells for photosynthetic cross-linking experiments, and the synthetic pre-S1 peptide was the key to identifying NTCP as a receptor for HBV and HDV [53]. Li et al. established a microRNA database for primary tree threw hepatocytes and analyzed the miRNAs from the primary hepatocytes of tree threw after HBV infection [54].

### HepaRG cells

In 2002, Gripon et al, researchers at the French National Institute of Medicine, isolated cells from the liver tumor tissues of female patients with HCV infection and secondary liver cancer. Initially, the cells obtained an undifferentiated morphology after several passages. Then, the authors induced the cells to differentiate into cells with the functional characteristics of mature hepatocytes and biliary cells by adding DMSO and hydrocortisone to the medium. Finally, through purification and screening, the cell line HepaRG was obtained [55]. HepaRG is a hepatic progenitor cell line with directed differentiation potential that has a morphology similar to that of mature hepatocytes and can express hepatocyte-specific proteins after induction. HepaRG cells have been confirmed to be infected with HBV and secrete HBV antigen particles as well as cccDNA. However, HepaRG cells are only partially sensitive to HBV. Schulze et al. analyzed the reason why HBV infection is dependent on the differentiation and polarization state of the cell. The formation of hepatocyte-like structures and the resulting transformation of membrane polarity render HepaRG cells susceptible to infection by allowing access to the basolateral localized HBV-specific receptor(s) [56]. This cell line can be used for transduction with adeno-associated virus (AAV) or lentiviruses and is also suitable for direct HBV serum infection similar to primary hepatocytes. HepaRG cells support the complete HBV life cycle, including the viral entry step, and are the best tool for HBV virology research and new drug screening [57]. When culturing these cells, DMSO was previously added to the medium to increase the infection rate of HBV [30]. However, despite improving the culture conditions, the efficiency of HepaRG infection with HBV was only approximately 10–15%. Moreover, as a cell culture system for studying HBV, the viral replication level in the HepaRG cell line is far less than that obtained with plasmid transfection. These issues have created bottlenecks for current *in vitro* infection experiments. Because this cell line can express various functionally normal detoxification enzymes, it has been widely used to study drug metabolism and toxicity [58, 59]. This cell line is also suitable for studying the mechanism of virus adsorption and entry into host cells under natural conditions.

### *In vitro* systems based on induced pluripotent stem (iPS) cell-derived human hepatocytes

Due to the scarcity of human primary hepatocytes and difficulties with long-term culture, the metabolic function of these cells is rapidly lost *in vitro* in short-term culture, which limits the use of primary hepatocytes. However, hepatoma cell lines lack a variety of cellular pathways and are not susceptible to HBV infection, making such cell lines unusable for the study of HBV-host interaction mechanisms. Therefore, it is necessary to find a more suitable cell culture system for studying the life cycle of HBV and the mechanisms of interaction with the host. Some researchers have attempted to use pluripotent stem cells as a breakthrough to establish cell culture systems suitable for HBV research. In 2007, the Japanese scientist Shinya Yamanaka transferred retroviruses containing human Oct3/4, Sox2, Klf4, and c-Myc into adult human dermal fibroblasts and dedifferentiated the cells into pluripotent stem cells by reprogramming. Induced pluripotent stem cells are similar to embryonic stem cells and embryonic adult pluripotent stem cells (APSCs), have multidirectional differentiation potential and are capable of maintaining genetic stability and self-renewal in culture [60]. Likewise, Duncan et al. produced human iPS (hiPS) cells by transducing foreskin fibroblasts with lentiviruses expressing OCT3/4, SOX2, NANOG and LIN28 [61, 62]. By supplementing the medium with B27 and 100μg/L activin A (ACTA), human iPS cells could differentiate into terminal endoderm, and continuously adding 20 μg/L BMP4, 10 μg/L FGF and 20 μg/L hepatocyte growth factor (HGF) under O2/5% CO2 culture conditions could further induce their directional differentiation into hepatocytes. HLCs derived from iPS cells have a morphology and characteristics similar to those of hepatocytes differentiated from human embryonic stem cells and have various liver function manifestations, including glycogen accumulation, indocyanine green metabolism, lipid accumulation, urea synthesis, uptake of low-density lipoprotein (LDL) and albumin expression. Mamoru Watanabe et al. established an immature proliferating hepatocyte-like cell line (iPS-HPCs) and a differentiated hepatocyte-like cell line (iPS-Heps) using induced pluripotent stem cell-derived hepatocyte lines. These two cell lines can express NTCP and their respective hepatocyte markers [63]. Sakurai et al. reported that with the differentiation of human-induced pluripotent stem cells into HLCs, the expression levels of several genes involved in HBV infection gradually increased [64]. Xia et al. Optimized the culture conditions to enable iPSCs to differentiate into HLC in a shorter time (15d). After differentiation, the HLCs maintained their differentiated state and allowed HBV infection for more than 4 weeks. Besides, they found that HLCs expressed the viral receptor NTCP more stable than primary human hepatocytes and HLCs supported robust infections and some spread of HBV [65]. Hideki Taniguchi et al. reported that HBV-infected human-induced pluripotent stem cell -derived liver organoids(hiPSC-Los) could recreate the virus life cycle and virus-induced hepatic dysfunction, which provides a promising individualized infection system for the development of personalized hepatitis therapy [66]. Notably, susceptibility to HBV infection in HLCs was largely dependent on the silencing of the type I interferon response which was demonstrated using a JAK inhibitor after infection. Because the innate immune system in hepatocytes derived from human pluripotent stem cells is intact, a cell bank containing iPS cell lines with extensive genetic variation could help elucidate virus-host interactions during chronic HBV infection and assist with drug development for HBV infection. The authors also confirmed that the expression level of NTCP is an important factor affecting the efficiency of HBV infection in iPS-HPCs. The HBV receptor NTCP, known as one of the central factors induced during the late differentiation of HLCs, was expressed at significantly higher levels in iPS-Heps than in iPS-HPCs, which explained why the shift point for becoming HBV permissive corresponded with the time of the phenotypic switch from hepatoblast-like to HLCs. The authors established the iPS-HPC-NTCP cell line by overexpressing NTCP, and these cells were shown to proliferate infinitely and to have biological characteristics similar to those of normal hepatocytes. The cells can stably proliferate over a long time while retaining a natural immune response. Therefore, they can be used to evaluate the effect of host cell maturity on the infectivity and life cycle of HBV and the effect of specific gene functions on host-HBV interactions. In addition, IPS-HPC and iPS-HPC-NTCP can be used for drug screening and studying the interaction between viruses and hosts and future genetic modifications in host cells. The shortcomings of this cell line are complicated modeling process, strict cell culture conditions and high experimental technical requirements.

### NTCP-overexpressing hepatoma cell lines

In general, the first steps in the viral infection of a host cell are binding to the surface receptors on the cell membrane and then entering the cell. Different viruses require their own receptors, and cells with the appropriate receptors can be effectively infected by viruses. Prior to the discovery of specific receptors, HBV was known to interact with cell surface heparan sulfate proteoglycan (HSPG) to mediate adsorption to susceptible cells, but this does not explain the biological mechanism that HBV specifically infects hepatocytes [67].

**Fig. 1:**
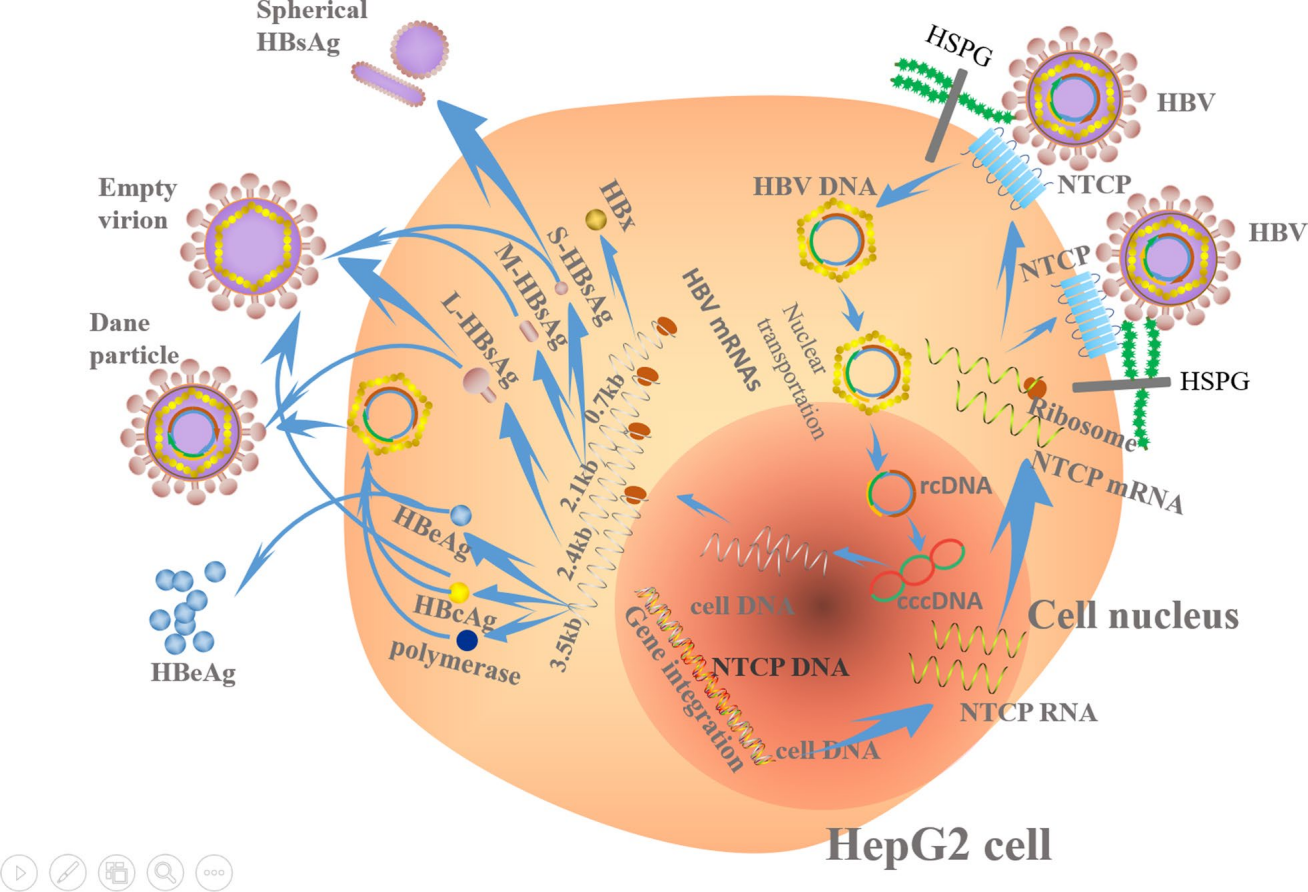
Schematic diagram of HBV entry into HepG2-NTCP cell mediated by NTCP. HBV interacts with the heparan sulfate proteoglycan on the cell surface and binds to the specific receptor NTCP which were overexpressed on the HepG2-NTCP cell, and then enters it. For detailed information, see the text. HSPG: heparan sulfate proteoglycan; NTCP: Na+-taurocholate co-transporting polypeptide; cccDNA: covalently closed circular DNA.

In 2012, Li et al. reported the discovery of NTCP, a specific receptor for HBV infection [53] (Fig. 1). The authors modified the peptide of the HBV binding receptor (Pre-S1, 2 to 48 amino acids) [68–72] as a probe to search for a protein that binds to the Pre-S1 peptide by using the near-zero distance cross-linking and affinity purification techniques, finally discovering NTCP. By using peptide competition, the authors verified that NTCP specifically bound to Pre-S1, and they then silenced the NTCP gene via molecular biological RNA interference, thereby reducing the infectivity of HBV and hepatitis D virus (HDV). All these findings demonstrate that NTCP is necessary for HBV and HDV infection (as shown in Fig. 1).The authors also discovered that HepaRG cells need to be differentiated by drugs for two weeks before being infected with HBV because of the increased levels of NTCP expressed by the cells after induction.

NTCP is a Na+ concentration gradient-dependent transporter located on the basolateral membrane of hepatocytes and can extract cholic acid from the blood [73, 74]. The NTCP expression in HepG2 and Huh7 cell lines, which are insusceptible to HBV, is relatively low, and the transporter is mainly expressed in hepatocytes, which are susceptible to HBV [75]. Destruction of the epithelial barrier of HepaRG cells grants HBV access to the basolateral membrane and thus increases the incidence of HBV infection. Unlike other epithelial cells, the location of NTCP on the basement membrane in mature hepatocytes depends on the polarity of the cell rather than the orientation of the apical membrane, which at least partially explains the problem of rapidly decreased HBV susceptibility caused by decreased primary hepatocytes polarity *in vitro* [56]. Yan H et al and Yi-Ni et al. compared human and mouse NTCP nucleotides and found that the sequence difference in the NTCP amino acid residues 84–87 was key to the species specificity of HBV infection by constructing a human mouse NTCP gene chimerism model. Replacing amino acids 84-87 in the mouse NTCP with the corresponding human sequence rescued the susceptibility of mouse hepatocytes to human HBV, suggesting that the second extracellular loop in mouse and human NTCP may be a crucial host determinant [72, 76]. Researchers have reported that NTCP-binding agents, including cyclosporin A (CsA) and its derivatives, as well as bile acids, can inhibit HBV entry by interrupting the interaction between NTCP and HBV large surface proteins [72, 77, 78]. Ro41-5253 was found to reduce host susceptibility to HBV infection by modulating the expression level of NTCP. All of these findings indicate that the regulatory pathway of NTCP expression is one determinant of HBV infection susceptibility [79]. Yan et al. used a plasmid as a vector to introduce the NTCP gene into Huh7 and HepG2 cell lines that cannot be infected by HBV or HDV and established HepG2-hNTCP and Huh7-hNTCP cell culture systems. After expressing the NTCP protein, these cells became susceptible to HBV and HDV. After transfected with NTCP, cell lines were infected with HBV, HBeAg, HBsAg, replication intermediates and RNAs could be detected in the culture supernatant [53]. In addition, cell lines such as hNTCP-HepaRG, hNTCP-HepG2 and hNTCP-HEK293 were created by transfection with the hNTCP gene. The discovery of NTCP made it possible to establish an HBV/HCV coinfection cell culture system that simulates natural infections. Yan et al. have demonstrated that overexpressing NTCP in HCV-susceptible Huh7 cells supports HBV infection. Veriier et al. discovered that NTCP mediates not only HBV infection but also HCV infection [80]. Generally, cell culture systems with high expression of hNTCP have the advantage of infinite hepatoma cells proliferations. After the first infection with HBV, virus particles secreted by cell lines with high expression of NTCP can still infect other cells, indicating that these models can support the whole life cycle of the virus and can be used to study the complete mechanism of HBV infection, including early viral invasion. Currently, these models have been used in the large-scale screening of antiviral drugs targeting NTCP [81–84]. However, these models disadvantageously require a high viral inoculum. Notably, Choijilsuren et al. observed that the physiological concentration of heparin could enhance HBV infection in an NTCP-dependent manner, leading to the establishment of a PEG-free HepG2-NTCP-AS platform that mimics HBV natural infection *in vivo* more closely than other systems [85]. However, this kind of model does not readily allow numerous viruses to spread between cells, which indicates that NTCP is not the only factor affecting HBV infection of host cells, even if it is possible to increase the infectivity of the progeny viruses by screening susceptible clones and changing the culture conditions [86, 87]. There are also other essential factors that influence HBV infection and replication and may be impaired or even lost in NTCP-overexpressing hepatoma cell lines. Despite the flexibility and handle ability of hepatocellular carcinoma cells, they lack multiple cellular pathways, including innate immune responses such as those related to IFN-α, which are particularly important for eliminating HBV from host cells. This limits their use in the study of virus-host interaction mechanisms [88, 89]. Therefore, although the establishment of an NTCP overexpression hepatoma cell culture system has made great progress, a more stable and more physiologically relevant system is still needed to mimic the HBV infection process *in vivo*.

## Conclusions

The *in vitro* HBV cell culture system is an important tool for screening anti-HBV drugs, studying the biological properties of HBV and investigating virus-host interactions. We summarized the advantages and shortcomings of all the cell culture systems (as shown in Table 1). Because of the host specificity and tissue specificity of HBV, the availability of a stable and reliable *in vitro* cell culture system for HBV research is a key factor affecting the study of the mechanism of HBV action. The existing HBV cell culture systems have played an important role in studying the pathogenesis of HBV infection, immune mechanisms, screening of anti-HBV drugs, etc. and have greatly promoted research on the biological characteristics, infection process, and pathogenesis of HBV as well as on the development of anti-HBV related drugs and vaccines.

**Table 1.** Summary of HBV *in vitro* hepatocyte culture models

Classification	Cell line	Advantages	Shortcomings	HBV infection rate and application of the models
HBV replication cell lines	(1) HepG2.2.15 cells	cccDNA accumulation Stable and continuous HBV gene expression and replication	Low viral replication level Antigen expression instability Virions are produced from the integrated DNA	Screening and evaluation of antiviral drugs, etc. [90].
	(2) HepAD38 (EF9,EFS19) cells	Cells differentiate quickly Produce high titers of viral particles cccDNA accumulation Hepatoma cells stably expressing HBV from a Tet-on/Tet-off system	Incomplete viral life cycle Virions are produced from the integrated DNA	Screening and evaluation of antiviral drugs, etc. A potential source for tissue culture derived virions [91].
	(3) Ad-HBV1.3-systems	No species barrier Efficient expression of HBV HBV expression and mutation can be controlled Direct observation of transfection and infection efficiency (integrated green fluorescent protein gene)	Missing HBV natural infection stage	Used to establish animal models of acute hepatitis B infection [92].
	(4) HBV baculovirus system	Easy detection of riboprotein-bound HBV DNA High HBV replication level Formation of infectious viruses and a detectable intracellular cccDNA pool	Nonreceptor-mediated entry Gene transfer is restricted to certain species Missing HBV natural infection stage	Quantify the effect of antiviral agents on nuclear HBV DNA Used for studying the resistance of HBV to nucleoside analogs [93].
Cell lines that can be infected with HBV	(1) Human fetal hepatocytes	Phenotypically and biologically functionally close to primary adult human hepatocytes	Low infection efficiency Short infection time Limited availability Large donor-donor variations	HBV infection rate12%-90% [22, 94]. Coculturing with hepatic non-parenchymal cells and subsequent addition of 2% DMSO leads to the formation of hepatocyte islands with prolonged phenotypic maintenance [25]. The early events in viral entry into cells as well as viral replication [23].
	(2) Adult human hepatocytes	The gold standard host cell to HBV infection experiments Closest to the physiological characteristics of hepatocytes *in vivo* Close to the natural process of infection	Limited life cycle Unpassable culture Phenotypically unstable *in vitro* Rapidly lose permissiveness for HBV infection Large donor-donor variations	HBV infection rate 20%-100% [26, 28]. Used for studying the process of HBV infection [5, 28]. Studying on apoptosis [26]. Preparation of 3D primary hepatocyte culture system for analyses of liver diseases, drug metabolism, and toxicity [40, 41].
	(3) Co-culture system	Test the utility of various direct-acting antivirals (DAAs) and putative host-targeting antivirals (HTAs); Assessing preclinically the efficacy of other entry inhibitors and possibly (vaccine-induced) neutralizing antibodies;	Wide variability between donors in terms of HBV permissiveness	Inflammation and drug-Induced Hepatotoxicity [95].
	(4) Primary Tupaia hepatocytes	The only species susceptible for HBV infection besides humans and chimpanzees	Expensive	HBV infection rate >70% [52]. Used for *in vitro* as well as*in vivo* infection experiments [96]. HBV specific receptor identification [78].
	(5) HepaRG cells	Preserve the specific functional properties of hepatocytes Support the complete HBV life cycle Produce HBV cccDNA	Strict culture conditions Low infection efficiency	HBV infection rate <30% [56, 78]. HBV molecular mechanism and screening, evaluation of anti-HBV drugs; cccDNA spread etc. [57]. Drug metabolism and toxicity [58, 59].
	(6) *In vitro* systems based on induced pluripotent stem (iPS) cell-derived human hepatocytes	Biological characteristics similar to those of normal liver cells Support the complete life cycle of the virus Complete natural immune system	Complicated operation	HBV infection rate 25% [97]. Drug hepatotoxicity screening [98]. The life cycle of HBV virus and virus-induced hepatic dysfunction [66].
	(7) NTCP overexpressing hepatoma cell lines	Support the complete life cycle of the virus Flexibility and easy handling	Low susceptibility to serum-derived HBV The multiplicity of infection (MOI) needed to achieve infection is extremely high No substantial viral spreading following infection	HBV infection rate 50% [99]. Large-scale screening of antiviral drugs for targeting NTCP [91].

Due to the presence of inhibitory factors in human serum, most HBV cell culture systems *in vitro* cannot be infected with HBV-positive serum. The HepG2.2.15 and HepAD38 cell lines can continuously secrete HBV particles because of the integration of the HBV genome. HepAD38 cells, in particular, secrete 11 times more virus than HepG2.2.15 cells and are often used as the source of virus for HBV infection in cell culture systems and widely used in related studies. HepG2.2.15 cells have been used in many laboratories to screen anti-HBV drugs. On the other hand, the discovery of the HBV receptor NTCP has promoted research on the mechanism of HBV infection. After overexpressing NTCP, some liver tumor cell lines that could not be infected with HBV became susceptible to HBV, and cell lines that could be infected by HBV, such as the HepaRG cell line, acquired increased susceptibility to HBV. However, cell culture systems that overexpress NTCP still do not result in high cell-to-cell spread and cannot simulate the natural processes of HBV infection. This observation also indirectly indicates that NTCP is not the only factor affecting HBV infection of the host, and tumor cell lines may not express the factors associated with HBV infection and replication. Comparatively, the most ideal model for studying the mechanism of HBV infection is human primary hepatocytes. However, their use is limited owing to the source scarcity and the inability to be cultured *in vitro* for a long period. In recent years, because of the rapid development of 3D culture technology, large-scale expansion of hepatocytes *in vitro* has become possible. A number of laboratories have reported a variety of 3D culture methods and the use of 3D culture technology to expand human primary hepatocytes *in vitro*. Although some of the reported 3D culture techniques have their own advantages and disadvantages, it is believed that in the near future, the further optimized culture system can lead to the achievement of large-scale human hepatocytes expansion *in vitro* and to the maintenance of mature hepatocyte function for a long period, thus providing an optimal model for the study of HBV infection. The advantages and disadvantages of various cell culture systems for HBV infection *in vitro* and their applications are shown in Table 1.

## Data Availability

Not applicable.

## Abbreviations

HBV: Hepatitis B virus
cccDNA: Covalently closed circular DNA
NTCP: Na^+^-taurocholate co-transporting polypeptide
GFP: Green fluorescent protein
MOI: Multiplicity of infection
KGF: Keratinocyte growth factor
VPP: Nicotinamide
ECGF: Endothelial cell growth factor
PEG: Polyethylene glycol
DMSO: Dimethyl sulfoxide
AAV: Adeno-associated virus
IPS: Induced pluripotent stem
hiPS: Human iPS cells
ACTA: Activin A
HGF: Hepatocyte growth factor
HLC: Hepatocyte-like cells
LDL: Low density lipoprotein
iPS-HPCs: Induced pluripotent stem cell-derived immature proliferating hepatic progenitor-like cell lines
iPS-Heps: Induced pluripotent stem cell-derived differentiated hepatocyte-like cells
hiPSC-Los: Human-induced pluripotent stem cell -derived liver organoids
HSPG: Heparan sulfate proteoglycan
CsA: Cyclosporin A
ECM: Extracellular matrix
ULA: Ultralow attachment
